# Exploring the Experience of Nurses in Providing Care to Patients With COVID-19: A Qualitative Study

**DOI:** 10.1097/jnr.0000000000000498

**Published:** 2022-05-17

**Authors:** Roonak SHAHOEI, Syede Mona NEMATI, Sina VALIEE

**Affiliations:** 1 PhD, Associate Professor, Clinical Care Research Center, Research Institute for Health Development, Kurdistan University of Medical Sciences, Sanandaj, Iran; 2 MSN, RN, Instructor, Faculty of Nursing and Midwifery, Kurdistan University of Medical Sciences, Sanandaj, Iran; 3 PhD, RN, Professor, Clinical Care Research Center, Research Institute for Health Development, Kurdistan University of Medical Sciences, Sanandaj, Iran.

**Keywords:** nurses, COVID-19, care, qualitative research

## Abstract

**Background:**

The COVID-19 pandemic has caused conflicts in the world health system. The role of nurses is prominent because of their close contact with patients.

**Purpose:**

The aim of this study was to explore the experiences of nurses in providing care to patients with COVID-19.

**Methods:**

This phenomenological study was conducted in 2020. Purposive sampling was used and continued until data saturation. Semistructured interviews were conducted with 14 nurses at Tohid Hospital, Sanandaj, Iran, all of whom had experience providing care to patients with COVID-19. Interviews were transcribed verbatim and analyzed using Colaizzi's phenomenological method.

**Results:**

The participants included four men and 10 women. Data analysis revealed four main themes (14 subthemes) related to the experience of the participants in providing care to patients with COVID-19. These themes included (a) fear (fear of being infected, fear of being a carrier, fear of the disease, and high mortality of patients), (b) compulsion (compulsion to care, being under pressure), (c) distinct experience (need for self-care, working with personal protective equipment, and ambiguity in care/treatment), and (d) sacrifice (altruism, compassion, self-sacrifice, and being proud of yourself).

**Conclusions/Implications for Practice:**

The experience of participants in caring for patients with COVID-19, in addition to the unique experience of care, was associated with fear, compulsion, and sacrifice. Because of the ongoing COVID-19 pandemic and the unique experience of nurses serving in COVID-19 units, it is necessary to educate and support nurses to deal effectively with this situation.

## Introduction

On December 29, 2019, hospital physicians in Wuhan, China, noticed unusual cases of patients with pneumonia. Studies have shown that the disease originated in the seafood, poultry, and live animal market in Wuhan City, Hubei Province in central China. Subsequently, an unusual outbreak of pneumonia was reported to the World Health Organization (Tavakoli et al., 2020). Since then, the disease has spread rapidly to other countries in a variety of ways, and the disease, severe acute respiratory syndrome coronavirus 2, or more informally called COVID-19, has become a worldwide pandemic (Kamrujjaman et al., 2020). Although patients may experience only mild symptoms such as cough, fever, headache, muscle aches, and gastrointestinal disorders, in some cases, it causes serious lung damage and death (Singhal, 2020). Low pathogenesis and high transmissibility are two unique features of this new virus that distinguish it from other members of the corona family such as severe acute respiratory syndrome (SARS) and Middle East respiratory syndrome (MERS; Jiang et al., 2020). COVID-19 has spread worldwide, including to Iran (Farnoosh et al., 2020). At the end of March 2021, the death rate for the disease worldwide was 1.4%. It has infected more than 123,000,000 people worldwide and resulted in the deaths of 2,700,000 people (Worldometers, 2021). COVID-19 has inflicted irreversible consequences on society and the healthcare system (Mohammadzadeh, 2020).

Today, COVID-19 is a global, life-threatening disease of international concern. Healthcare workers, especially nurses, have close contact with infected patients and play an important role in controlling infection (Choi et al., 2020). Nurses comprise the largest group of healthcare providers and are the mainstay of the care process (World Health Organization, 2016). Nursing professionals are able to effectively respond to the numerous challenges facing the healthcare system. However, the shortage of nursing staff has been strongly felt, and the fatigue and exhaustion of working nurses have challenged healthcare systems (Heidari et al., 2012).

During natural disasters and infectious and contagious disease outbreaks, nurses sacrifice their personal needs to actively care for those in need and make selfless contributions that reflect their sense of moral and professional responsibility (Aliakbari et al., 2015). Concurrently, nurses experience mental and psychological stresses and feel isolated and helpless facing health threats and stressful work caused by natural disasters and infectious and contagious disease outbreaks. Previous studies have shown that nurses in close contact with patients with emerging infectious diseases such SARS (Chung et al., 2005), MERS (Khalid et al., 2016; Kim, 2018), Ebola virus disease (C. Liu et al., 2019), and Influenza A (H1N1; Honey & Wang, 2013) experience fear, fatigue, sleep disorders, and other physical and mental health problems. The rates of depression, insomnia, and posttraumatic stress disorder in nurses involved in providing care to patients with SARS were 38.5%, 37%, and 33%, respectively (Su et al., 2007). However, in a study on the psychological status of caregivers of patients with Ebola, 29% of respondents felt loneliness and 45% reported seeking psychological counseling (Smith et al., 2017). In a study focusing on the psychological experience of nurses caring for patients with COVID-19, it was found that initially negative emotional care prevailed and gradually positive emotions appeared (Sun et al., 2020).

Exploring the experience of nurses in caring for patients is important. As COVID-19 is a new disease and the facilities and approaches of medical systems differ from country to country, research on the experience of frontline nurses in the fight against COVID-19 is necessary. Most published studies highlight the prevalence of this disease and its clinical features, diagnosis, and treatment (Fan et al., 2020; Huang et al., 2020). Some studies have reported an increased incidence of severe physical and psychological problems in medical personnel (Kang et al., 2020; Xiang et al., 2020). However, few qualitative studies on the experiences of nurses have been published. Therefore, this study was designed to explore the experiences of nurses in providing care to patients with COVID-19.

### Context of the Study

In Iran, most hospitals and healthcare centers are owned and operated by the national healthcare system, which is administered by the Ministry of Health, Treatment & Medical Education (Ahmadi Chenari et al., 2020). The number of active beds in Iran is nearly 120,000, which translates into roughly 1.5 beds per 1,000 population (Haghdoost et al., 2018). In Iran, around 180,000 nurses work in the national healthcare system (World Health Organization, 2021), with more than half working in public hospitals, some 12% working in military hospitals, 12% working in social security hospitals, 6% working in private hospitals, and 6% working in prehospital emergency services (Heidari et al., 2012).

## Methods

### Design

A phenomenological approach was used to explore the experience of nurses providing care to patients with COVID-19 during 2020.

### Participants and Sampling

Nurses who worked in the specialized COVID-19 wards in Tohid Hospital in Sanandaj, Iran, were recruited using purposive sampling. Inclusion criteria were nurses who had provided care for patients with COVID-19 and were willing to participate. Exclusion criteria were nurses who were unwilling to participate or continue participating in the study. The sampling continued until data saturation was reached (Holloway & Galvin, 2016). Fourteen participants were interviewed.

### Data Collection

Data were collected using semistructured face-to-face interviews. Before the beginning of the interviews, potential participants were recruited by creating a trust-based atmosphere by explaining the purpose of the research and ensuring the confidentiality of information. The recorded interview started with open-ended questions. Participants' nonverbal behaviors and changing moods were documented. Demographic information, including age, gender, education, marriage, childbearing, work experience, caregiver ward, and work history, were collected after the interview. The interview questions and prompts were the following:

- Please explain how you provide nursing care to patients.
- Please explain your experience of providing care to patients with COVID-19.
- What is the difference between providing care to patients with COVID-19 and caring for other patients?
- What does caring for a patient with COVID-19 look like to you?

Data were collected from May to June 2020. The interviews took place in a private room at the hospital to ensure privacy. The length of each interview was between 30 and 90 minutes. The interviews were conducted and analyzed in Kurdish and only translated into English for the final report. Each participant was interviewed 1 or 2 times. The second interview, if needed, was conducted to fill in gaps in information from the first interview, do a member check, and resolve possible ambiguities. The recorded interviews were transcribed verbatim.

### Data Analysis

Data analysis was performed using Colaizzi's seven-step phenomenological method. The steps of this method are (a) familiarization, (b) identifying significant statements, (c) formulating meanings, (d) clustering themes, (e) developing an exhaustive description, (f) producing the fundamental structure, and (g) seeking verification of the fundamental structure (Morrow et al., 2015).

The researchers analyzed the data according to the following steps: repeatedly read all transcriptions to capture the participants' experiences, extracted statements related to the desired phenomenon (the process of extracting the main statements), extracted the concept and meaning of each of the main statements that were extracted in the previous step, organized the concepts formulated into specific thematic categories, combined all the extracted ideas into a complete and comprehensive description of the phenomenon under study, turned the comprehensive description of the phenomenon into an explicit/unambiguous description, and clarified with participants that research findings were valid and reflected their actual views.

### Rigor

Four criteria, including credibility, dependability, conformability, and transferability (Thomas & Magilvy, 2011), were used to ensure rigor. The researchers were actively involved with participants; chose an appropriate research approach; gained familiarity with the existing context of the participants; selected a diverse sample; performed member checking, peer checking, and expert checking; and used direct quotes to support the findings. The interviewer summarized information and then questioned the participants to determine accuracy. Member checks were undertaken during the interview process and at the end of data analysis. After data analysis, the findings were shared with the participants. For peer checking, impartial peers examined the study's approach, transcripts, codes, and themes. To verify the obtained findings, the viewpoints of a professor of nursing were gained regarding the final reported findings. The corresponding author conducted and transcribed the interviews and analyzed the data. All of the steps were checked by the members of the research team, of which all members are experienced qualitative researchers.

### Ethical Considerations

This study was approved by the Research Council of the Clinical Care Research Center (No. 1399.052) and the ethics committee of Kurdistan University of Medical Sciences (No. IR.MUK.REC.1399.052). All of the participants were volunteers and provided signed informed consent. The study purpose was clarified to the participants, and they were assured about the confidentiality of their conversations and that related audio files would be deleted after transcription. Participants were assured that they could withdraw from the study at any time, even after providing informed consent. All of the participants' names were changed into codes during the transcription process, and the same codes were used in data analysis and the report.

## Results

The participants in this study included four male and 10 female nurses, of which five were married and nine were single. One of the female nurses had a child, and another was pregnant. Furthermore, one participant lived alone, whereas others lived with two, three, or four family members (mean = 2.78, *SD* = 0.97). The religion of all participants was Islam. Nine of the participants worked in the intensive care unit, and the remaining five provided care services in the general ward. The nurse–patient ratios in the intensive care unit and general wards were 1:3 and 1:7, respectively. The mean age of participants was 29.28 (*SD* = 5.7) years, the average length of service was 5.35 (*SD* = 4.37) years, and the average length of service in the COVID-19 ward was 1.6 (*SD* = 0.74) months. Moreover, the average amount of work was 219.42 (*SD* = 20.13) hours per month. Five participants did not have any experience in infectious disease care, whereas nine had an average of 2.77 (*SD* = 2.16) months of experience. Finally, eight participants had received training on providing care in specialized COVID-19 wards.

The initial data analysis revealed four main themes and 14 subthemes: (a) fear (fear of being infected, fear of being a carrier, fear of the disease, and high mortality of patients), (b) compulsion (compulsion to care, being under pressure), (c) distinct experience (need for self-care, working with personal protective equipment (PPE), and ambiguity in care/treatment), and (d) sacrifice (altruism, compassion, self-sacrifice, and being proud of yourself; Table 1). In the subsequent data analysis, all of the emergent themes were collapsed into one, exhaustive description that described the comprehensive structure of the phenomenon. This description was: “The experience of nurses in providing care to patients with COVID-19 was a unique experience involving fear, sacrifice, engagement, in despite feelings compulsion to care and being under pressure.” Thereafter, the researchers sought a qualitative expert researcher who reviewed the findings to confirm that this exhaustive description reflected the experience of nurses providing care to patients with COVID-19. Finally, a validation to this exhaustive description was confirmed with the research team.

**Table 1.** Summary of Categories and Subcategories

Theme	Subtheme
1. Fear	(1) Fear of being infected (2) Fear of being a carrier (3) Fear of the disease (4) High mortality of patients
2. Compulsion	(1) Compulsion to care (2) Being under pressure
3. Distinct experience	(1) Need for self-care (2) Working with personal protective equipment (3) Ambiguity in care/treatment
4. Sacrifice	(1) Altruism (2) Compassion (3) Self-sacrifice (4) Being proud of yourself

In the sixth step, the exhaustive description was condensed into a short, dense statement meant to capture only those aspects deemed to be essential to the structure of the phenomenon. The fundamental structure of the nursing experience providing care to patients with COVID-19 included three parts: event, emotional reaction, and support as “sacrifice in the situation of fear and compulsion.”

Finally, the study findings were validated via member checking, which was undertaken by returning to the participants and discussing the findings with them. Participants' views on the study findings were collected via WhatsApp. This step was done by the main researcher who had interviewed the participants. All of the participants expressed satisfaction with the findings, believing them to accurately reflect their experiences.

### Fear

The first theme extracted from the interview was fear, which included fear of being infected, fear of being a carrier, fear of the disease, and fear of the high mortality of patients. One aspect of “fear” in participants was the fear of being infected. Thus, they were afraid of getting COVID-19 from patients while providing care. For example, one participant noted that “...*when providing patient care, one feels fear of the possibility to become infected*...” (P3), and another participant said, “...*I am really stressed and worried for my own health first when it comes to prioritizing my own or the patient's health*...” (P8). Another aspect of the “fear” theme was the fear of being a carrier. They were afraid to transmit the disease to their family. For example, one of the participants mentioned that “…*I was very afraid to pass the disease onto my family, especially my child, because they announced that children could also be infected*…”(P1), and another participant said, “...*I'm really afraid of passing the disease onto my parents as they are seniors*...” (P13). Fear of the disease was another part of the first theme. Participants were afraid of COVID-19 because the disease was novel and its dimensions were unknown. A nurse with 13 years of work experience said, “...*the high prevalence of this disease and its rapid onset also led to a sense of fear in us*...” (P5), and another nurse said, “...*The novel aspects of this disease and treatment methods are also frightening*...” (P7). The high death rate of patients was one of the most important manifestations of the participants' fears: “...*Because I did not get a definite answer from anyone, I felt frustrated and the patients’ deaths bothered me a lot*...” (P11) and “...*A large number of patients died at great cost, which was very hard for me to accept...*” (P9).

### Compulsion

Another important part of the nurses' experience in providing care to patients with COVID-19 was “compulsion,” which included the subthemes of being forced to care for patients and being under pressure. The participants were forced to provide care in COVID-19 patient wards: “...*At first, I did not like caring for Corona virus patients; however, I was forced (or required) to go to the corona ward*...” (P8) and “...*Because the hospital had become a corona center, it became mandatory to provide care to these patients*...” (P9). Another aspect of compulsion was the pressure on the nurses. Because of the high number of hospitalized patients and the high rate of mortality, the participants felt significantly more pressure compared with the situation before the outbreak of COVID-19: “...*It was very difficult to take care of these patients*…” (P10), said one of the participants, and another participant said, “...*The patients did not seem to suffer, but they died after a few days, and that was really hard for me*...” (P6).

### Distinctive Experience

The third theme identified was distinctive experience, which included three subthemes: need for self-care, working with PPE, and ambiguity in care/treatment. One of the distinctive aspects of caring for these patients was the need for self-care. The participants felt an increased need for self-care because of the increased need for protection while providing care. One of the participants stated that “...*the difference between caring for a Corona patient and other patients relates to the ward and self-care*...” (P8), and another participant said, “...*Patient care is very different from previous patient care practices because here we have to be very careful and we must observe the necessary protection as much as possible*...” (P3). Another important aspect of the participants' experiences was working with PPE. Participants said that wearing and working with PPE was a new experience that was different from the usual care they provided: “...*The problem is wearing PPE for the Corona patients, which is kind of a new situation for us*...” (P10), “...*We used to take care of patients very easily, but, in the Corona ward, we have to wear special clothes and follow many hygiene guidelines*...” (P6), and “...*One of the differences of caring for Corona patients is personal clothing and protective equipment, which makes it really difficult to work with*...” (P4). Ambiguity in care/treatment was another manifestation of the participants' experiences. Lack of information and ambiguity in providing care and treatment for patients with COVID-19 made a different experience for participants of providing care: “...*Providing care to a Corona patient is completely different because we were not very sure about the treatment ourselves*...” (P5), “...*What can I do if there is an unpredicted problem with the patient, it was a new experience*...” (P7), and “...*that we do not really know what to do for the patient and that there is no clear protocol or clear result, has made patient care different from other patients*…” (P11).

### Sacrifice

The fourth identified theme was sacrifice, which included four subthemes: altruism, compassion, self-sacrifice, and being proud of yourself. All of the participants expressed a sense of altruism when sharing their experiences. They choose to work with patients with COVID-19 out of altruism and trying to improve their patients' well-being: “...*After a while of providing care to COVID-19 patients, I was very happy to help them*...” (P9) and “...*In general, I think there is a sense of altruism in all of us that makes us take care of these patients*...” (P4). Another case of sacrifice was the experience of a sense of self-sacrifice. The participants, despite knowing that working in the COVID-19 ward endangered their lives, emphasized the health of their patients: “...*Exactly from the first day I went to the Corona ward, I was sure that I would risk my life But, because of that sense of sacrifice and self-sacrifice, I accepted it*...” (P3) and “...*In a situation where everyone cares about themselves, I care about my fellow human beings and their health is important to me and a sign of my self-sacrifice and devotion*...” (P5). Compassion was a feeling that most of the participants experienced while caring for these patients. The experience of the participants was accompanied with feelings of sympathy and concern for the sufferings of the patients and their families: “...*I feel deep sympathy for patients when I see them in a state where they have no companions or visitors. I really feel sorry for them*...” (P14) and “...*On the other hand, when one sees how much they are afraid and worried about the disease, one feels compassion for the patient*...” (P3).

## Discussion

This study was designed to explore the experience of nurses in providing care to patients with COVID-19 using a phenomenological method. The findings showed that the experience of the participants in caring for patients with COVID-19 encompassed fear, compulsion, distinct experience, and sacrifice.

### Fear

Fear was an important part of the participants' experiences. Threats to personal health, lack of awareness, and being at risk of catching a disease can lead to negative emotions such as fear, worry, and anxiety, which have been reported in other studies (Khalid et al., 2016; Kim, 2018; O'Boyle et al., 2006). The participants in this study were afraid of COVID-19 and considered caring for infected patients to be a threat to their own health. Xiong and Peng (2020) also reported that healthcare providers feared infection and the potential of infecting others. Other studies indicate that nurses who provided care during infectious disease outbreaks feel the most pressure and, at the same time, fear the disease (Lam & Hung, 2013; Sun et al., 2020). One study in Saudi Arabia revealed a high level of fear and anxiety about MERS among medical students (Al-Rabiaah et al., 2020), which was consistent with the results of this study. Possible causes of this fear and concern may be infection, difficulties faced in controlling the epidemic, and a lack of adequate medical equipment.

Another cause of fear was the fear of being a carrier, which was consistent with a study by S. H. Lee et al. (2005). Nurses are most afraid for the older adults and for their families and children (S. H. Lee et al., 2005). During care, it is important to establish basic supportive systems, including the provision of appropriate PPE, reasonable allocation of human resources, care services for the older adults and children of nurses' families, and prework training to facilitate nurses' adaptation to work under pandemic conditions (Chung et al., 2005). Facing a high mortality rate among patients was another cause of fear in the participants. One of the relevant features of the current COVID-19 pandemic is the unpredictability in terms of the numbers of critically ill patients and high mortality rates, which increase the risk of posttraumatic stress disorder among healthcare workers and the general public (Carmassi et al., 2020; Z. Li et al., 2020). Factors such as family and social support, support from supervisors and colleagues, training, work organization, and coping strategies help improve resilience in facing the COVID-19 pandemic (Carmassi et al., 2020). Therefore, supporting nurses at the individual, family, social, and organizational levels is necessary. For example, ensuring suitable organizational support, increasing social support, providing psychological and mental support facilities, and providing stress management and resilience promoting interventions are recommended (Labrague & De los Santos, 2020). Because of the fear expressed by the participants, supportive measures to eliminate the causes of this fear, including appropriately distributing shifts among nurses and offering special consideration and arrangements for nurses with underlying conditions and for those who are pregnant or have older adults or young children in their family, may be helpful in this regard.

### Compulsion

Being forced to care for patients and being under pressure elicited similar compulsion experiences among the participants. During the SARS and MERS epidemics, healthcare providers also reported psychological experiences and were at a higher risk of mental health problems (S. M. Lee et al., 2018; Marjanovic et al., 2007). Nurses' experiences of care for patients with MERS in South Korea were accompanied by “going into a dangerous field” (Kim, 2018). In Sun et al. (2020), nurses stated that they were forced to perform high-intensity work for patients with COVID-19 (Sun et al., 2020), which was consistent with the results of this study. The participants in this study felt pressured and experienced difficult conditions. Other studies have shown that caring for a patient infected with an infectious agent creates difficult situations (Lam & Hung, 2013; Schwartz et al., 2014) but that tolerating these difficult situations leads to an increased capacity to accept responsibility, preparing nurses to accept greater responsibility and, if a similar situation occurs in the future, to be good supporters. In contrast, the results of a study by Sun et al. showed that most nurses were under physiological stress and experienced less stress and difficulty of work conditions (Sun et al., 2020). The results of this study are similar to those in the literature examining the experiences of nurses caring for patients with H1N1, SARS, and Ebola in terms of difficulty and frustration with patient treatment and death (Lam & Hung, 2013; Smith et al., 2017). Despite the difficult conditions and compulsory care, the participants in this study expressed that they were unable to refuse hospital requests because of the chronic lack of professional manpower and their feelings of professional responsibility. Torda (2006) also found that nurses behaved ethically and continued to provide care when necessary (Torda, 2006). Nurses are productive when they provide quality care. Therefore, reducing work-related stress by supporting and responding to the needs of nurses, making their shifts voluntary in COVID-19 wards when possible, and permitting additional vacation and welfare benefits to reduce their stress may be necessary.

### Distinctive Experience

Similar to other new infectious diseases such as Ebola, there was initially no effective drug to treat COVID-19 and nurses provided the first line of care (Fan et al., 2020). However, healthcare providers in other sectors had little clinical experience in infectious intensive care. When healthcare systems are not prepared to deal with the spread of an infectious disease and treatment and care protocols are unclear (L. Li et al., 2020), training and improving communication are essential. According to Alipour et al. (2020), the unknown nature of COVID-19 and cognitive ambiguity are sources of anxiety in individuals, which is consistent with the results of this study.

In addition to regularly providing care to patients, the necessity of using protective equipment for long hours also leads to physical distress, especially in nurses who must stay in isolation wards for their entire shift (Adams & Walls, 2020). The COVID-19 crisis led to healthcare providers engaging in intensive work for many hours while wearing PPE (Fan et al., 2020). Because of the high prevalence of this disease, it is necessary to take care of the healthcare staff, especially nurses. When healthcare workers become ill, concerns are raised about their ability to control the spread of the disease and treat patients. Therefore, it is important for authorities to set minimum working hours, arrange reasonable shifts to support healthcare providers, and protect them against overwork. There is a clear need to explore the aspects of every new COVID-19 nursing caring experience. Therefore, authorities should interact and communicate with these nurses and facilitate this new experience for them as much as possible with the goal of minimizing the possible future consequences for nurses.

### Sacrifice

Sacrifice was the final theme identified in this study. The findings of various studies indicate that a sense of responsibility based in professional ethics encourages nurses to actively participate in antipandemic work and strengthens their altruism and professional pride (Aliakbari et al., 2015; Khalid et al., 2016). In this study, the participants expressed feeling useful and altruistic when providing care to patients and were proud of themselves. However, these results were not consistent with the results of some other studies, in which nurses expressed deep negative feelings when attending to the care needs of inpatients (Kang et al., 2020; Xiang et al., 2020). Nevertheless, other studies have reported similar findings (Honey & Wang, 2013; H. Liu & Liehr, 2009). Because most of Iran's population is Muslim, most nurses are religious and often incorporate their religious beliefs, which emphasize empathy, into their care responsibilities (Ghaljeh et al., 2016). When care is based on compassionate behaviors, the patient may more easily express the symptoms of their disease and their concerns (Zamanzadeh et al., 2016). Therefore, in situations in which patients do not have companions or visitors and the outbreak of this new disease invokes concern, compassion may help nurses accomplish their patient care responsibilities.

Because patients with COVID-19 are quarantined, feel frightened and anxious (Chen et al., 2020), and experience limited interpersonal communication, they experience negative emotions (Xiao, 2020). This leads nurses to feel pity when providing care to these patients. The nurses in this study expressed feeling an extraordinary sense of responsibility and strong willingness to sacrifice personal safety and interest for teamwork to treat patients with COVID-19. Positive emotions such as self-sacrifice play an important role in healing and regulating psychological damage (Waugh, 2014). Therefore, strengthening multidimensional social support, adjusting cognitive evaluation, guiding positive coping styles, and stimulating positive emotions are very important to promoting and improving the psychological health of nurses. Strengthening this feeling in nurses in the current situation can improve their tolerance for future, new conditions.

Overall, the participants in this study experienced both positive and negative emotions during their care of patients with COVID-19. At first, because of their lack of knowledge about and high prevalence of the disease, negative emotions such as fear prevailed. However, as time passed and with the experience of caring for these patients, positive emotions increased in prominence. Despite the difficult conditions and high pressure, the nurses coped with all of these problems, did their best to provide quality care, and were proud of this sense of responsibility. Healthcare providers are highly resilient and self-sacrificing because they know they must be strong and focus on their job to save patients' lives. Reflecting the experiences of nurses to the authorities and the people may further support nurses and follow the instructions at the community level.

The experience of the participants in this study in caring for patients with COVID-19 shared similarities and differences with the general experience of nurses in various situations within Iranian medical and cultural contexts. A similarity was the sense of sacrifice among nurses. Generally, the nursing experience in Iran has been reported as being altruism oriented. Iranian nurses think that the nature of nursing practice is based on sacrifice and that caring for patients is what makes the nursing profession attractive to them (Nikbakht Nasrabadi et al., 2003).

Another aspect of the similarities/differences of the care experience was the uniqueness of the caring experience. The experiences of caring can differ significantly by context such as end-of-life care (Valiee et al., 2012), cancer care (Borhani et al., 2013), and care provided during conflict (Firouzkouhi et al., 2013). However, in this study, need for self-care, working with PPE, and ambiguity in the care or treatment of patients made each nurse's experiences unique. Providing care with PPE, like wearing a mask and wearing protective clothes, is similar to Iranian nurses' experiences working in chemical emergency departments during the Iran–Iraq war (1985–1988). Moreover, Iranian nurses' experiences providing care during conflict also involved self-sacrifice (Firouzkouhi et al., 2013).

In another aspect, participants were required to work shifts in COVID-19 wards, which increased their perceived stress. The nursing shortage had increased the pressure on Iranian nurses even before the COVID-19 outbreak (Shamsi & Peyravi, 2020). However, the COVID-19 pandemic increased this pressure further by requiring nurses to take care of numerous hospitalized patients with COVID-19 and face the increased rates of patient mortality.

The main difference between the findings of this and prior studies was the fear reported by the participants. No previous study has identified fear as a significant experience of Iranian nurses in taking care of patients. In the experience of nurses in other countries and cultures, fear has been reported in the care of patients with infectious diseases (Al-Rabiaah et al., 2020; Kim, 2018). Therefore, considering the new and unknown features of COVID-19, it seems normal that nurses are afraid of infection or transmission to others. However, in providing care for noncommunicable diseases, this feeling does not exist.

Finally, COVID-19 is a life-threatening illness with a global prevalence that is of international concern. The disease was first reported in Wuhan, China. Because of the outbreak of the virus, people in more than 220 countries are now infected and the epidemic of the virus is a global emergency. Healthcare workers, especially nurses, have a close relationship with infected patients and play an important role in controlling the outbreak. In Iran, which has one of the top 20 highest rates of infection worldwide, exploring the experience of nurses in providing care to patients with COVID-19 can be an effective step in controlling and improving the condition of nurses. Although the context of this study may differ from other studies, it seems that nurses' experiences in caring for patients with COVID-19 are highly similar because of similar pandemic conditions in other parts of the world. Thus, during this pandemic, supporting this important group in the healthcare team is critical.

### Limitations and Suggestions

Because of the nature of qualitative research, the sample size used in this study was limited. A larger sample taken from a broader population of nurses may reflect more diverse points of view. This study was conducted on nurses only, and the experiences of other staff and healthcare providers may be examined in future studies. A further limitation of this study was the transference of concepts into the local language. Interviews were conducted and interpreted in Kurdish. The English translation of the article was checked by Kurdish language experts. Because of disease prevention and control protocols, focus group discussions were not possible in this study and, to control infection risk, data were collected from one center only.

### Conclusions

The findings of this study provide a deep understanding of the experience of nurses in providing care to patients with COVID-19 using a phenomenological approach. During the COVID-19 pandemic, the positive and negative emotions of frontline nurses were found to be intertwined. In addition to work-related fears and pressures, the participants expressed feeling responsible and tried to provide the best, most compassionate treatment to their patients. Therefore, supporting nurses to handle their healthcare responsibilities under pandemic conditions while avoiding burnout is essential.
